# Chemical Composition and Future Perspectives of Essential Oil Obtained from a Wild Population of *Stachys germanica* L. Distributed in the Balkan Mountains in Bulgaria

**DOI:** 10.1155/2023/4275213

**Published:** 2023-10-31

**Authors:** Stanislava Ivanova, Stela Pashova, Stanislav Dyankov, Yoana Georgieva, Kalin Ivanov, Niko Benbassat, Nina Koleva, Maria Bozhkova, Diana Karcheva-Bahchevanska

**Affiliations:** ^1^Department of Pharmacognosy and Pharmaceutical Chemistry, Faculty of Pharmacy, Medical University-Plovdiv, Plovdiv 4002, Bulgaria; ^2^Medical College, Medical University-Plovdiv, Plovdiv 4002, Bulgaria

## Abstract

*Stachys germanica* L. (*Lamiaceae*) is a plant associated with a rich history in the traditional medicine of Iran, Turkey, and Serbia. However, researchers have not fully investigated the pharmacological potential of the herb, and scientific data on this plant species are limited. The aim of the current study was to evaluate the chemical composition of the essential oil (EO) obtained from the aerial parts of *S. germanica* L. growing wild in Bulgaria and to perform a comparative analysis of the chemical composition of EOs obtained from the same plant species from other geographical regions. For the evaluation of the chemical profile of the isolated EO, gas chromatographic analysis with mass spectrometry was performed. The most abundant terpene class was oxygenated monoterpenes, which accounted for 59.30% of the total EO composition. The bicyclic monoterpene camphor, as a compound of this class, was identified as the major constituent in the EO, accounting for 52.96% of the total oil composition. The chemical profile of Bulgarian EO is quite different compared to that of EOs from other regions. It is the only one to contain more than 50% camphor. In addition, EO contains significant amounts of the diterpene geranyl p-cymene (10.49%). This is the first study describing the chemical composition of EO from Bulgarian *Stachys germanica* L., and our results reveal some future perspectives for the evaluation of the biological activity of EO from the studied plant species as a new therapeutic agent or natural remedy targeting different medical conditions. The EO has a promising potential to be used as a biopesticide and repellent as well, an environmentally friendly and safer alternative to standard pesticides.

## 1. Introduction

*Stachys germanica* L., also known as Downy Woundwort, and German hedgenettle, belongs to the genus *Stachys* of the plant family *Lamiaceae* [1, 2]. *S. germanica* L. is a perennial plant with well-defined basal rosettes and leaves that are densely covered with long, velvety silvery hairs [3, 4]. It can reach a height of up to 120 cm [1]. The flowers exhibit shades of purple or pink [5]. This plant is commonly found in various habitats, including forests, bushy areas, sunny slopes, rocky and rugged sites, and meadows at elevations of up to 1400–1500 m [3, 6, 7].

The genus *Stachys* L. (*Lamiaceae*) unites about 300 species [1, 8]. Members of this genus are associated with a nearly worldwide distribution and could be found in regions of the Mediterranean, southern Africa, Asia, North, and South America [1, 8]. There are 20 naturally occurring species of *Stachys* in Bulgaria, some of which are specific only for the Balkan Peninsula [9].

The name of the genus originates from Ancient Greek; the word “stachys” (*στ*ά*χυς*) means “spike of corn” [8].

Some of the most important species of the genus are: *S. alpina* L., *S. officinalis* L., *S. recta* L. subsp. *recta*, *S. recta* L. subsp. *subcrenata* (Vis.) Briq., *S. obliqua* Waldst. et Kit., and *S. germanica* L. [1].

Plants belonging to this genus are rich sources of various bioactive compounds, possessing different biological activities [4, 8, 10–27]. For millennia, plants belonging to this genus have been used in the traditional medicine of different nations for the treatment and management of medical conditions such as spleen sclerosis, inflammatory conditions, nervous disorders, cough, stomach issues, skin problems, and infected wounds [4, 8, 10, 11, 13, 15, 20, 22, 23, 25–29]. Additionally, they have been recognized for their psychotropic effects, anxiolytic properties, and muscle relaxant activity [5, 15]. Some representatives have been observed to impact human metabolism as well, displaying potential antidiabetic and antiobesity activity [30].

Based on the data of traditional medicine and some recent studies, plants of the *Stachys* species could be promising sources of novel herbal medicines, dietary supplements, or functional foods [27, 31–33].

For example, the use of *S. germanica* L. in traditional medicine is well documented: in Iranian folk medicine the plant is used to alleviate dysmenorrhea and gastrodynia [28, 34]. In Turkey, *S. germanica* is commonly brewed into tea due to its antibacterial properties [27]. In Serbia, tea derived from *S. germanica* is popularly used for the treatment of respiratory issues [35].

Regardless of the data based on folk medicine, scholars have not fully investigated the pharmacological potential of the herb. However, there are some reports of active remedial effects associated with this plant. Some studies suggest that the plant possesses an anti-inflammatory activity that may assist in reducing inflammation and associated symptoms [11, 27]. Moreover, extracts obtained from the plant are believed to exhibit antioxidant activity that could help diminish the presence of oxidative stress within the human body [36]. According to several studies, extracts of *S. germanica* demonstrate promising antimicrobial potential, displaying the ability to inhibit the growth of pathogenic bacteria and fungi [27, 37].

While specific *in vivo* studies on *S. germanica* are limited, there are some *in vitro* studies conducted on this plant that demonstrate anticancer and antioxidant activity [20, 35].

Similar to numerous other plants belonging to the *Lamiaceae* family, *S. germanica* L. is a source of essential oil (EO) [1].

The main compounds identified in the EO include germacrene-D, *β*-caryophyllene, and *β*-farnesene [4, 18, 21, 22, 38]. The EO has been reported to exhibit antifungal, antibacterial, and antioxidant activities [18, 21, 35].

However, the chemical composition of the EO could be significantly affected by the geographical location, collection time, harvest year, and the growth stage of the plant as well. When reviewing the literature, no records were found about the composition of *S. germanica* L. EO from Bulgaria.

The focus of the present study was the evaluation of the chemical composition of the EO obtained from aerial parts of *Stachys germanica* L. growing wild in the Balkan region of Bulgaria and to compare it with EOs from different geographical regions.

## 2. Materials and Methods

### 2.1. Chemicals and Reagents

For determination of the retention indices (RI), C9–C20 n-alkane series (99% purity) purchased from Merck KGaA (Darmstadt, Germany) were used. The EO was diluted with hexane (≥97.0% purity), Sigma Aldrich (Steinheim, Germany) prior to the GC-MS analysis.

### 2.2. Plant Material and Oil Extraction

Aerial parts of *Stachys germanica* L. were carefully collected during the flowering period of the plant in June 2022 from the region of Gabrovo (42°49′39.7″N 25°18′17.6″E), Central Balkan Mountains of Bulgaria. The taxonomic identity of the plant was determined according to Delipavlov and Cheshmedzhiev [9].

The EO was isolated from the aerial parts (stems, leaves, and flowers) of air-dried Stachys germanica L. (100 g) by hydrodistillation for 4 hours using a Clevenger-type apparatus. The next step was drying the EO over anhydrous sodium sulphate and stored in dark glass vials at 4°C before analysis. The percentage oil yield was determined on the basis of the dry weight of the plant.

### 2.3. Chromatographic Conditions

The composition of the obtained EO was evaluated using gas chromatography with mass spectrometry (GC-MS). The analysis was performed using a Bruker Scion 436-GC SQ MS (Bremen, Germany) equipped with a Bruker BR-5 ms fused silica capillary column (0.25 *μ*m film thickness and 15 m × 0.25 mm i.d.). As a carrier gas, helium was used. The flow rate was constant- 1 mL/min, with a linear velocity of 51 cm/s. The volume of injection was 1 *μ*L with an injector split ratio of 1 : 10. The oven temperature was initially set at 50°C for 1 min, then increased to 160°C at a rate of 3°C/min, and then increased to 270°C at a rate of 15°C/min and held for 2 min. The injector's temperature was set to 250°C, and the detector temperature was set to 300°C. The mass spectra were collected in full scan mode with a mass range of 50–350 m/z. The identification of the compounds of the EO was achieved by comparing their MS spectra and retention indices (RI) with spectral data within the Wiley NIST11 Mass Spectral Library (NIST11/2011/EPA/NIH) and the literature data [39]. According to the retention times of the C9–C20 n-alkane series injected under the same conditions described above, the RI values of the determined compounds were calculated.

## 3. Results and Discussion

### 3.1. Volatile Constituents of Essential Oil of *S. germanica* L. Grown Wild in Bulgaria

Hydrodistillation of the aerial parts of *S. germanica* L. gave yellow oil with a yield of 0.05% (v/w).

**Figure 1:**
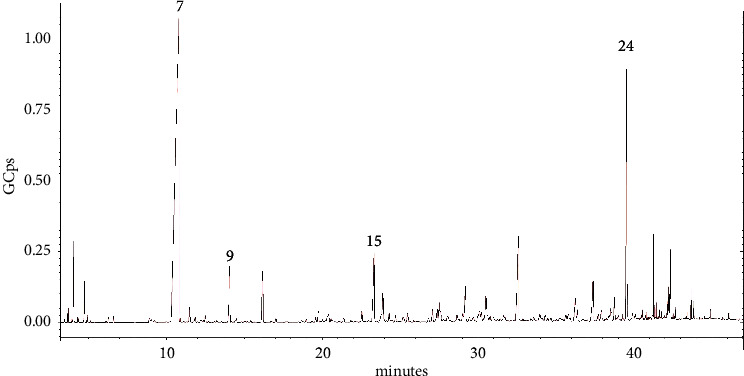
GC-MS chromatogram of the *S. germanica* EO, where GCps–giga counts per second, and the numbers refer to the following compounds: 7-camphor, 9-trans-chrysanthenyl acetate, 15-(E)-*β*-farnesene, and 24-geranyl-p-cymene.

The composition of the EO was evaluated using GC-MS. The chemical analysis resulted in the identification of twenty-four volatile compounds, representing 84.82% of the total EO. The most abundant terpene class was the oxygenated monoterpenes, which accounted for 59.30% of the total EO. The main compound of this class was camphor, which was the main compound identified in the EO, overall, representing 52.96% of the total oil composition. In addition, the EO contained significant amounts of the diterpene geranyl-p-cymene (10.49%). Sesquiterpenes were identified in smaller amounts. The principal sesquiterpene identified in the EO was the sesquiterpene hydrocarbon (E)-*β*-farnesene (3.97%). The rest of the detected sesquiterpenes were less than 2% (*α*-copaene, *β*-elemene, caryophyllene, germacrene-D, *α*-muurolene, *δ*-cadinene, nerolidol, spathulenol, caryophylene oxide, and *α*-cadinol).  Figure 1 and Table 1 show the chromatogram and the data obtained by the GC-MS analysis.

### 3.2. Comparison of the Main Volatile Constituents of the EO of *S. germanica* L. from Different Regions

The following table (Table 2) compares the main components found in EOs obtained from *Stachys germanica* L. from different geographical locations. Although the harvesting regions varied, the data collected indicated the presence of almost identical main components–germacrene-D, *β*-farnesene, and *β*-caryophyllene. However, the chemical profile of Bulgarian EO is quite different. In comparison to EOs from other regions, it is the only one which contains more than 50% camphor. Essential oils from the studied plant species distributed in Macedonia also showed the presence of camphor, but in significantly smaller amounts, about 10-fold less [38].

Within the content of Bulgarian EO, geranyl p-cymene emerges as the second most abundant substance, found at a concentration of 10.49%, whereas it is not mentioned among the major compounds of the other countries considered.

Despite variations in factors such as harvest periods and region, the main constituent consistently observed in EOs from Greece, Hungary, Serbia, Italy, and Turkey is germacrene-D [18, 21, 22, 34, 42]. Germacrene-D, as a sesquiterpene, is the dominant compound found in Italian EO extracted from leaves (39.40%), whereas its presence in EO obtained from flowers is significantly lower (12.80%) [34]. This is closely followed by the species harvested in Turkey, with a concentration of 27.10% [22]. Germacrene-D is not only known for its unique aroma, often described as spicy and pungent, but is also associated with diverse biological activities [28, 34, 44, 45]. It has demonstrated antitumor effects against different cancer cell lines, antimicrobial activity, and skin recovery effects [20, 28, 46]. At the same time, the compound has promising potential for use as a biopesticide [47, 48]. Germacrene-D is either absent or present in negligible quantities not only in Bulgarian EO but also in EOs derived from certain Serbian and Italian representatives of *Stachys germanica* L. [4, 43]. Similary to germacrene-D, caryophyllene oxide is also present in plants from many countries such as Greece, the Former Yugoslav Republic of Macedonia, Hungary, and Turkey [21, 22, 38, 42]. The concentration of this substance varies from 13.40% to 0.57%. However, in Bulgarian EO, its concentration is even lower, 0.23%. Caryophylene oxide belongs to the group of oxygenated sesquiterpenes and exhibits a diverse spectrum of pharmacological effects, including antibacterial, antifungal, anti-inflammatory, and antioxidant properties, it is considered as a therapeutic candidate for the prevention and treatment of cancer as well [49–51].

Bulgarian EO contains minimal amounts of *β*-caryophyllene, which is one of the major compounds found in Turkish species, where the concentration fluctuates around 15% [22]. *β*-Caryophyllene is classified as a sesquiterpene hydrocarbon and is associated with antibacterial, antifungal, and anti-inflammatory activity [21, 28, 42, 43].

In addition to the general compounds mentioned earlier, each country also has its own specific set of compounds. For example, Bulgaria is notable for its remarkable concentration of camphor, while Italy demonstrates compounds such as (Z, Z, Z)-9,12,15-octadecatrienoic acid and epi-bicyclosesquiphellandrene [4]. Some compounds occur in several countries but in different concentrations. Included in this group are bicyclogermacrene D and spathulenol. These compounds are present in EOs of *Stachys germanica* L., collected in Greece (2.4%) and Serbia (2.4%, 8.97%) for bicyclogermacrene, and in Turkey (3.7%, 5.8%) and Serbia (4.6%) for spathulenol [18, 21, 22, 48]. Bicyclogermacrene has demonstrated exceptional effectiveness in eliminating mosquito larvae [52]. On the other hand, spathulenol has shown the ability to restrain the growth of lymphocytes [53]. This compound has also been examined for its effectiveness against mycobacterial infections [54, 55]. When applied topically, it notably suppresses pain sensitivity [56].

### 3.3. Future Perspectives for the Use of Bulgarian *Stachys germanica* L. Essential Oil

Nowadays, many contemporary pharmaceutical roots could be found in the traditional medicine of different nations [57]. Although the pharmaceutical revolution has led to the synthesis of numerous new molecules, many significant novel medicines are expected to have natural origins and to be based on data from folk medicine [44, 57, 58].

The main findings of this study suggest that there are opportunities for future application of the EO derived from Bulgarian *Stachys germanica* L. However, more studies are needed for the evaluation of the biological activity and safety of this EO. Due to its exceptional antioxidant properties, Bulgarian *S. germanica* L. EO has the potential to serve as a raw material in the pharmaceutical and cosmetic industries. In contrast to earlier investigations, the major compound found in Bulgarian *S. germanica* L. EO was *camphor* 52.96%. Camphor is a bicyclic monoterpene which has a wide range of pharmaceutical applications [59, 60]. This constituent is naturally present in *Cinnamomum camphora* [45, 61]. It was originally obtained from this species by distillation, but now it is produced synthetically from turpentine. Camphor is also found in various aromatic plant species, including *Lavandula stoechas*, *Cinnamomum tamala*, *Eucalyptus globulus*, *Salvia officinalis*, *Salvia glutinosa*, *Artemisia annua, Tanacetum parthenium*, *Tanacetum vulgare*, *Tanacetum armenum*, and *Ocimum canum* [47, 62–66]. Camphor has been administered as a central nervous system stimulant, an analeptic, cardiac, contraceptive, cold remedy, as well as an insect repellent [67]. It is used topically as a pain reliever to treat skin diseases such as itching, eczema, and fungal infections [60, 68–70]. Furthermore, the compound is considered an excellent skin penetration enhancer [71]. Camphor is also associated with antimicrobial, antiviral, antinociceptive, antitussive, and anticancer activities [71–73]. In addition, camphor acts as a local anesthetic by inhibiting skin sensory receptors [68]. Camphor finds application in the topical treatment of joint inflammation and back pain as well [59].

However, the high levels of camphor (52.96%) make the Bulgarian *Stachys germanica* L. EO unsuitable for oral intake or other internal administration. Nevertheless, toxicity tests more often relate to single pure compounds than to mixtures. Essential oils are natural complex substances. This makes them particularly challenging as they are not only mixtures, but different batches/sources contain different concentrations of the components tested. In the application of an essential oil, interactions may be between one or more of its constituents. The interactions between the constituents in a blend are very difficult to predict, from additivity through synergism to antagonism. In the context of pharmacology, this would mean that the therapeutic dose could be reduced. However, from a toxicology point of view, the enhanced action would be undesirable, and while this may be adverse to the therapeutic effect, it would be favourable for toxicity [74]. In addition, camphor containing EOs have a promising potential to be included in ointments or other products for topical application to treat the symptoms of conditions such as myalgia, arthritis, pain, and inflammation.

Another noteworthy characteristic of camphor is its repellent activity [75]. Nowadays, there is a great demand for biopesticides as environmentally friendly and safer alternatives to conventional pesticides. Several studies have already demonstrated the efficiency of some EOs and their phytoconstituents in pest management [76–79]. Because of its chemical profile, the EO of *S. germanica* L. has also demonstrated significant potential for application as a biopesticide and repellent [76].

The levels of *β*-farnesene in Bulgarian EO were 3.97%. This compound is a lipophilic sesquiterpene associated with anti-inflammatory activity in human neutrophils [80]. It also exhibits insecticidal activity and acts as a pheromone in some insects, particularly bees [81]. Although the levels of *β*-farnesene in Bulgarian EO are not high, in combination with the high levels of camphor (52.96%), synergism in anti-inflammatory activity could be observed.

In the EO extracted from Bulgarian *Stachys germanica* L., bornyl acetate was identified as a component comprising 2.54% of the oil`s composition. This bicyclic monoterpene exhibits a diverse array of biological activities and is generally considered as safe [82, 83]. The application of bornyl acetate is mainly associated with its anti-inflammatory effects [82–84].

Zhao et al., suppose that the bornyl acetate anti-inflammatory mechanism of action is based on interfering with the classical inflammatory signal pathways, NF-*κ*B and Mitogen-Activated Protein Kinase (MAPK), by inhibiting phosphorylation of I*κ*B, JNK, p38, ERK, and reducing secretion of TNF-*α*, IL-1*β*, and IL-6 [83]. Bornyl acetate exhibits a nitric oxide inhibitory activity as well, which is also beneficial for the anti-inflammatory activity [83].

Inhalation of bornyl acetate induces a gentle sedative effect [85]. Furthermore, the compound possesses anticancer activity, antimicrobial activity, and exhibits hypotensive effects [83, 86]. It also finds application in cosmetics as an agent with antioxidant and antispotting efficiency [87]. Bornyl acetate is known for its insecticidal activity as well, which enables it to be used as an environmentally friendly repellent [88, 89]. Although the compound presents in low concentrations in this EO, it could potentiate the anti-inflammatory and the analgesic effects of camphor. However, the synergism between these molecules should be better studied.

**Figure 2:**
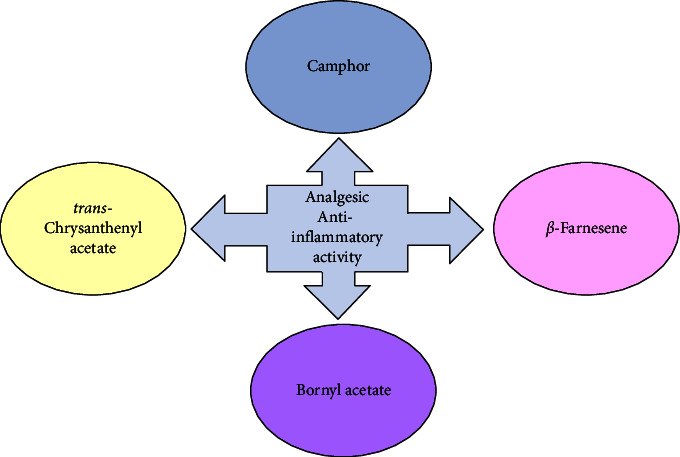
Future perspectives for the use of Bulgarian *Stachys germanica* L. EO.

Trans-Chrysanthenyl acetate, which was found to be present in the EO at a concentration of 2.66%, is also a compound with analgesic and antimicrobial activity [90, 91]. It has been reported as the predominant compound in EOs extracted from various plant species across different families [91]. It exhibits phytotoxic properties against harmful weeds [92]. Trans-chrysanthenyl acetate has been identified as the dominant compound in the EOs of other plant species which also exhibit phytotoxic efficiency [93, 94]. Another characteristic associated with this compound is its allelopathic activity [91, 94]. The phytotoxic properties of this compound make it highly relevant for potential use in agricultural applications and for the synthesis of naturally derived herbicides. Although the compound is in low concentrations, it could provide synergism along with camphor, *β*-farnesene, and bornyl acetate in terms of the analgesic and anti-inflammatory activity of the EO (Figure 2). The combination of these molecules could be especially beneficial for topical application for the treatment/relief of joint or muscle pain in acute or chronic conditions.

Although camphene does not present in significant amounts in the Bulgarian EO–2.52%, it could provide some beneficial effects and even synergism together with camphor, trans-chrysanthenyl acetate, and *β*-farnesene. This compound is classified as a monoterpene hydrocarbon, which occurs in different fruits, nuts, and EOs [95–98]. It has been extensively used as a flavor enhancer and a fragrance component in the cosmetics and food industries [99]. Studies have revealed that camphene exhibits a range of biological activities including antibacterial and antifungal effects, antioxidant, and anti-inflammatory properties [100–104]. In addition, camphene is widely recognized for its insecticidal and larvicidal activities [105–107].

Geranyl-p-cymene, which has been found in the EO at concentrations of about 10%, might be biologically relevant to the insect-plant relationship, although the available data are inconclusive [108]. This compound has been identified in EOs from two Turkish endemic species-*Stachys tmolea* subsp. *tmolea* Boiss. and *Stachys cretica* subsp. *trapezuntica* Rech. [109, 110]. However, further research is required to elucidate the precise importance of geranyl-p-cymene in the complex dynamics of insect-plant relationships.

Future *in vitro* and *in vivo* studies would be especially beneficial in providing a more robust understanding of the future application potential of the Bulgarian *Stachys germanica* L. EO. Nevertheless, the chemical profile of the EO indicates external use only.

Novel products based on different EOs are highly likely to be introduced in the cosmetics, agriculture, and pharmaceutical industries in the next few years. EOs are not only sources of compounds with biological activity but also unique compositions of molecules that have the potential for serious synergism [111–114].

## 4. Conclusions

This is the first report on the chemical profile of EO isolated from Bulgarian *Stachys germanica* L. Our results revealed the presence of twenty-four volatile compounds including camphor, geranyl-p-cymene, (E)-*β*-farnesene, and trans-chrysanthenyl acetate. The present study highlights the potential applications of the essential oil in the pharmaceutical industry. In comparison to EOs derived from other geographical regions, this EO stands out as the only one that contains more than 50% camphor, which has a wide range of pharmaceutical applications. However, the high levels of camphor (52.96%) make the Bulgarian *Stachys germanica* L. EO unsuitable for oral intake or any other form of internal administration. Nevertheless, the studied EO exhibits promising potential to be incorporated into pharmaceutical products for topical application to treat various symptoms and conditions such as myalgia, arthritis, pain, and inflammation. The synergistic effects between the compounds could enhance the analgesic and anti-inflammatory activities of the EO. Furthermore, the chemical profile of the EO is associated with its potential utilization as a biopesticide. Future studies would be especially beneficial in providing a more robust understanding of the future application perspectives of Bulgarian *S. germanica* L. EO.

## Figures and Tables

**Table 1:** Volatile constituents of the EO of *S. germanica* L. from Bulgaria as a percentage of total EO.

No.	Compound	RI	RI lit. data	Formula	Class of compound	% of total EO
1	*α*-Pinene	957	944	C_10_H_16_	MH	0.55
2	Camphene	966	956	C_10_H_16_	MH	2.52
3	Benzaldehyde	972	968	C_7_H_6_O	O	0.17
4	*β*-Pinene	982	980	C_10_H_16_	MH	1.32
5	1-Octen-3-ol	987	986	C_8_H_16_O	O	0.16
6	Trans-*β*-Ocimene	1034	1038	C_10_H_16_	MH	0.17
7	Camphor	1144	1144	C_10_H_16_O	MO	52.96
8	Borneol	1159	1156	C_10_H_18_O	MO	0.59
9	Trans-Chrysanthenyl acetate	1224	1228	C_12_H_18_O	MO	2.66
10	Bornyl acetate	1277	1273	C_12_H_20_O_2_	MO	2.54
11	*α*-Copaene	1366	1372	C_15_H_24_	SH	0.43
12	*β*-Elemene	1379	1387	C_15_H_24_	SH	0.53
13	*β*-Caryophyllene	1403	1411	C_15_H_24_	SH	0.18
14	Octyl 2-methylbutanoate	1433	1436	C_13_H_26_O_2_	O	0.45
15	(E)-*β*-Farnesene	1457	1458	C_15_H_24_	SH	3.97
16	Germacrene-D	1471	1470	C_15_H_24_	SH	0.9
17	*α*-Muurolene	1490	1488	C_15_H_24_	SH	0.25
18	*δ*-Cadinene	1510	1509	C_15_H_24_	SH	0.42
19	Bornyl angelate	1550	1547	C_15_H_24_O_2_	MO	0.55
20	(E)-Nerolidol	1558	1561	C_15_H_26_O	SO	0.39
21	Spathulenol	1562	1582	C_15_H_24_O	SO	0.95
22	Caryophylene oxide	1564	1565	C_15_H_24_O	SO	0.23
23	*α*-Cadinol	1638	1639	C_15_H_26_O	SO	1.44
24	Geranyl-p-cymene	1898	No data	C_20_H_30_	O	10.49

	*Terpene classes*					
	MH—monoterpene hydrocarbons					4.56
	MO—oxygenated monoterpenes					59.30
	SH—sesquiterpene hydrocarbons					6.68
	SO—oxygenated sesquiterpenes					3.01
	O—others					11.27
	Total identified					84.82

The relative percentage of peak area is shown as the mean of three independent measurements and the standard error of the mean has been removed and does not exceed 2%. ^*∗*^A comparison was made between the RI obtained for each compound and the reference values from the literature data [40, 41].

**Table 2:** Comparison of the main volatile constituents of the EO of *S. germanica* L. from different regions.

Plant collecting region	Main volatile compounds %	Other volatile compounds	Ref.
Bulgaria	Camphor (52.955%) geranyl-p-cymene (10.49%)	*β*-Farnesene (3.97%) trans-chrysanthenyl acetate (2.66%) bornyl acetate (2.54%) camphene (2.52%)	Present study
Greece^1^	Germacrene-D (21.3%) (−)-(E)-caryophyllene (18.4%)	*δ*-Cadinene (6.8%) (−)-*α*-copaene (6.6%) (+)-caryophyllene oxide (5.9%) dodecanal (5.3%) bicyclogermacrene (2.4%)	[21]
Former yugoslavian republic of Macedonia^1^	(E)-Nerolidol (13.5%) caryophyllene oxide (13.4%)	Germacrene-D (8.1%) camphor (5.7%) *β*-caryophyllene (5.1%) valeranone (4.8%) (E)-*β*-farnesene (3.1%)	[38]
Hungary	Ethyl hexadecanoate (32.73%) germacrene-D (7.32%)	*β*-Phellandrene (4.76%) trans-*β*-farnesene (1.98%) sabinene (1.48%)	[42]
Serbia	Borneol (9.27%) bicyclogermacrene (8.97%)	*β*-Farnesene (5.7%) spathulenol (4.6%) humulene epoxide (4.24%) farnesene epoxide (4.08%) 2,3-dimethyl-2-butanol (4.07%)	[43]
Serbia	Germacrene-D (16.49%) phenyl ethyl heptanoate (13.75%) (E)-*β*-farnesene (13.26%)	2-Acetyl-naphthalene (7.21%) (Z)-*β*-ocimene (5.02%) (+) *β*-pinene (4.28%) octyl 2-methyl butyrate (2.8%) bicyclogermacrene (2.4%)	[18]
Italy	(Z,Z,Z)-9,12,15-octadecatrienoic acid (33.3%) hexadecanoic acid (22.1%)	Epi-Bicyclosesquiphellandrene (6.1%) 4-vinylguaiacol (3.4%) cis-*β*-farnesene (3%) *α*-bisabolol (3%) tetradecanoic acid (2.5%)	[4]
Italy^2^	Germacrene-D (39.4%) phytol (10.2%)	*β*-Bourbonene (3.5%) *β*-ylangene (3.3%) hexadecanoic acid (3.2%)	[34]
Italy^2^	Limonene (24.1%) *β*-pinene (18.7%) germacrene-D (12.8%)	(E)-Nerolidol (6.6%) *β*-ylangene (4.4%) *β*-elemene (3%)	[34]
Turkey^1^	Germacrene-D (27.1%) *β*-caryophyllene (15.7%) caryophyllene oxide (12.8%)	*τ*-Muurolol (4.8%) *α*-cadinene (3.9%) spathulenol (3.7%) *α*-copaene (3.2%) geranyl acetate (3.1%)	[22]
Turkey^3^	Germacrene-D (23.2%) *β*-caryophyllene (14.8%)	*α*-Copaene (7.7%) caryophyllene oxide (6.9%) trans-*β*-farnesene (6.8%) spathulenol (5.8%) *α*-cadinene (4.9%)	[22]

^1^ *S. germanica* L. ssp. *heldreichii*; ^2^*S. germanica* L. ssp. *salviifolia*; ^*3*^*S. germanica* L. ssp. *bithynica*.

## Data Availability

All data generated and analyzed during this study are included in the manuscript.
